# Evaluation of Oleogels Stabilized by Particles of Birch Outer Bark Extract through a Novel Approach

**DOI:** 10.3390/gels9110911

**Published:** 2023-11-17

**Authors:** Sanita Vitolina, Rudolfs Berzins, Janis Rizhikovs, Daniela Godina, Zoltán Márk Horváth, Konstantins Logviss, Arturs Teresko, Aigars Paze

**Affiliations:** 1 Biorefinery Laboratory, Latvian State Institute of Wood Chemistry, LV-1006 Riga, Latvia; rudolfs.berzins@kki.lv (R.B.); janis.rizikovs@kki.lv (J.R.); daniela.godina@kki.lv (D.G.); aigars.paze@kki.lv (A.P.); 2 Laboratory of Finished Dosage Forms, Faculty of Pharmacy, Riga Stradiņš University, LV-1007 Riga, Latvia; zoltanmark.horvath@rsu.lv (Z.M.H.); konstantins.logviss@rsu.lv (K.L.); 3 ZS DOKTUS, LV-4101 Cesis, Latvia; arturs_teresko@inbox.lv

**Keywords:** birch outer bark extract, betulin, oleogel, vegetable oil, oil structuring, rheology, gel strength

## Abstract

Structuring liquid oils into oleogels using natural and abundant biomass components as gelling agents is of great significance in fields such as foods, pharmaceuticals, and cosmetics. In this work, a more energy-efficient and technologically simpler approach for directly preparing birch outer bark extract particles containing oleogel was used. This method involves introducing birch outer bark extract particles into the oil phase directly from the extract solution, combining both the evaporation of solution and gel formation. As a result, stable oleogels of various vegetable oils (sunflower, almond, olive, and hemp seed) were obtained with birch outer bark extract particle contents of 4–10%. Infrared spectroscopy and particle size analysis confirmed that when increasing the content of extract particles in the oil, increasing self-assembly of extract particles via hydrogen bonding occurs, leading to the formation of a more structured network. This is consistent with gel strength values from rheological tests of oleogels, which also increased with particle concentration. The obtained oleogels showed important properties such as good tolerance to time-dependent deformation, shear thinning, and thermoreversibility.

## 1. Introduction

Structuring liquid oils into gel-like solid materials (oleogels) has attracted much attention due to the application of extended functionalities in the fields of food, pharmaceuticals, and cosmetics [1]. Oleogels can be formed from a variety of gelators, such as polysaccharides, proteins, or low-molecular-weight self-assembling molecules, e.g., monoglycerides, fatty alcohols, fatty acids, waxes or phytosterols, and their esters [2]. However, the increasing trend, determined by environmental considerations and consumer awareness, to use natural materials in various formulations and the need for safe, efficient, low-cost, and abundant gelators is still a driving force for new research [3,4].

The outer white layer of birch (*Betula* spp.) bark has been extensively studied for its potential to be processed into valuable compounds [5,6,7]. Birch outer bark is, by virtue of its chemical composition, a unique natural product containing a large amount (~35 wt.%) of valuable extractives, the majority of which (~75 wt.%) is the triterpene group compound betulin. Apart from betulin, birch outer bark also contains other very valuable compounds that are not harmful to the normal cells of the human body, such as lupeol, betulinic acid, and phenolics. It has been well established that these compounds can be extracted in good yields (20–40 wt.%) from birch outer bark with a great number of different organic solvents [6].

Several studies have shown that naturally occurring pentacyclic triterpenes, such as betulin, lupeol, and betulinic acid, can spontaneously self-assemble in liquids to form supramolecular structures [8,9,10]. For example, birch outer bark extractive micro/nanoparticles obtained by liquid antisolvent precipitation after filtration form a hydrogel-like structure with a high liquid content (up to 97 wt.%) [11,12,13]. A similar self-assembly for extractive particles has also been described in an oil medium. Purified birch outer bark dry extract obtained by high-pressure solvent extraction exhibits the ability to form an oleogel when it is suspended in oil [14]. The obtained formulations are sufficiently stable that no additional additives, such as preservatives or emulsifiers, are needed. The gelation mechanism was studied more closely by Grysko [15], and the formation of a three-dimensional network, which was stabilized by the hydrogen bonds of the surface carboxyl and hydroxyl groups, was found. The resulting network immobilized the lipophilic liquid. The interaction between the extract particles and the hydrogen bonds plays a crucial role in the formation of the gel network. Furthermore, the factors affecting the gelation mechanism also include the concentration of extractives, the specific surface area of the particles, and the type of lipid phase [16]. If water is also incorporated into the oleogel stabilized by the extract, W/O creams can be obtained [17]. In addition to the interesting gelling properties, birch outer bark extract also exhibits numerous pharmacological properties [18]. The antibacterial effects of the extract are of great importance for the preservation of semi-solid preparations [19,20,21]. The extract also exhibits anti-inflammatory activity [22,23,24] and properties that promote wound healing [25,26,27]. Together with their pharmacological effects, the obtained birch outer bark extract particle gels, where extractives act simultaneously as a biologically active ingredient and as a gelling agent, are of special significance due to their potential range of applications in pharmacology, cosmetics, and food industry.

Birch outer bark extracts for use in the production of semi-solid preparations are currently obtained by pressurized liquid extraction (PLE) with organic solvents like n-heptane [28,29] and supercritical fluid extraction (SFE) using supercritical carbon dioxide as an extraction agent [30]. To our knowledge, there are no reports on the stabilization of semi-solid systems using a solution of unpurified birch outer bark extract obtained via the classical solid–liquid extraction method using boiling solvents under atmospheric pressure and directly introducing them into the lipophilic phase in the form of extract particles without separating them via evaporation and drying. Obtaining a birch outer bark extract solution by extraction in boiling solvents requires technologically less advanced and, accordingly, less expensive extraction equipment than is the case for PLE and SFE [31]. The direct use of the extract solution without an additional drying step to obtain semi-solid systems makes this technique more energy-efficient. Also, the energy consumption required to operate high-pressure equipment for the same working volume could be higher than that when operating at atmospheric pressure.

In this context, the main objective of the study was to obtain stable oleogels using a more energy-efficient and technologically simpler approach for direct oleogel production by adding the birch outer bark extract particles to the oil phase from solution, combining both solution evaporation and gel formation [32]. The effect of the extract particle content in sunflower oil on the gelling ability and the properties of the obtained oleogels were investigated and characterized. Furthermore, the ability of the extract particles to structure other vegetable oils (olive, almond, and hemp seed) with this new approach was also evaluated.

## 2. Results and Discussion

### 2.1. Characterization of Obtained Oleogels

**Figure 1:**
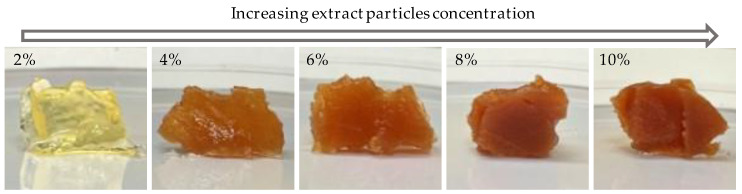
The concentration effect of the extract particles on the appearance of sunflower oil oleogels.

The ethanol solution of birch outer bark extract served as the raw material for preparing oleogels based on sunflower oil, with varying concentrations of extract particles (2, 4, 6, 8, and 10 wt.%). Using our novel approach to introduce birch outer bark extract particles into the oil phase, we successfully obtained oleogel samples with a solid-like appearance. Figure 1 displays images of the obtained oleogels prepared at different concentrations of birch outer bark extractives in sunflower oil. It is noticeable that the oleogel with the lowest particle content (2%) differs significantly in color from those formulated with higher contents. In the case of the 2% particle concentration sample, the gel structure did not develop immediately but only after approximately 24 h. During this period, chromophoric compound particles could be observed settling to the bottom of the container, resulting in the obtained gel having a light yellow oil color. This sample also exhibited slight oil droplet separation during storage (over 1 month), indicating a weaker oil-binding capacity.

The content of betulin and two additional triterpenes (lupeol and betulinic acid) in the obtained oleogel samples was analyzed using GC-FID analysis. The data presented in Table 1 reveal that the betulin content of the total extractives in the obtained oleogels falls within the range of 46.8–50.5 wt.%, which is notably lower than the content reported in the literature for similar gels. Grysko [16], in his studies involving extract powders obtained through high-pressure solvent extraction with a high betulin content (>80 wt.%), demonstrated that the triterpene composition significantly influences the properties of birch outer bark extractives. The gel-stabilizing ability of the extractives is primarily attributed to betulin, and insufficient betulin content could negatively impact gel formation. However, our results indicate that using the direct particle introduction technique from the extract solution into the oil phase allows for the successful structuring of liquid sunflower oil into a soft solid material, even with unpurified extractives. These oleogels exhibit the same total particle concentration in the oil as in Grysko’s study (≥2%) but possess a significantly lower betulin content.

**Figure 2:**
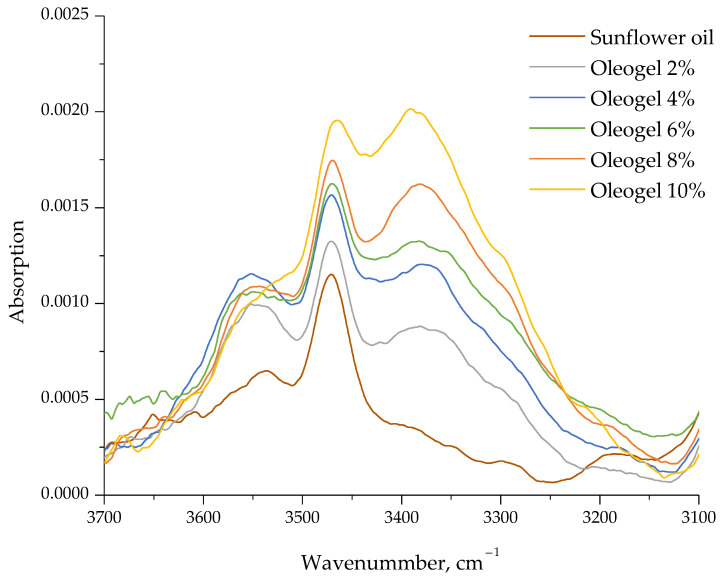
FTIR spectra of sunflower oil and obtained oleogels prepared with different concentrations of extract particles.

Colloidal gels consist of dispersed particles that interact with each other to create a network [33]. Previous literature suggests that an oleogel containing birch outer bark extract particles is formed through the self-assembly of these particles, stabilized by noncovalent interactions, primarily hydrogen bonding [15]. Van der Waals and electrostatic interactions, in addition to hydrogen bonds, may also contribute to the oil gelation [34]. Hydrogen bond formation between the extract particles in the obtained oleogel samples was analyzed by FTIR spectroscopy. FTIR spectra in the range of 4000–500 cm^−1^ for different oleogel samples showed similar peak positions, indicating no chemical interaction. Spectral differences were observed only in the low absorption bands associated with the OH groups. Figure 2 magnifies the spectral range 3700–3100 cm^−1^ in the FTIR spectra of sunflower oil and oleogels with varying birch outer bark extract particle concentrations. In the sunflower oil spectrum, peaks at 3470 and 3534 cm^−1^ are attributed to the OH vibration of the free hydroxyl groups due to the presence of compounds such as terpenoids, polyphenols, and sterols. The introduction of birch outer bark extract particles into the oil phase caused an increase in the intensity of the OH bands. Hydrogen bonding between the OH groups widened and strengthened the infrared absorption peak, shifting it toward the lower frequencies, resulting in a new absorption peak with a maximum of 3361–3378 cm^–1^. The absorption band in this region increases with higher extract particle concentration, indicating an augmentation of intermolecular hydrogen bonds and the formation of a presumably stronger gel network.

**Figure 3:**
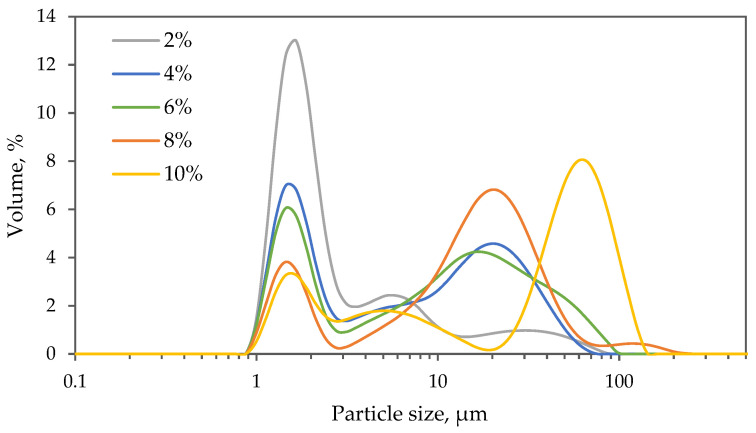
Effect of birch outer bark extract particle concentration on particle size distribution in sunflower oil oleogel.

Evaluation of birch outer bark extract particle size in the obtained oleogel samples was conducted using laser diffraction. All samples exhibited a bimodal particle size distribution (Figure 3), featuring one fraction between 0.9 and 3 µm and another fraction between 3 and 200 µm. The specific distribution within these fractions varied based on the particle concentration in the oleogel. As the particle concentration increased, the volume percentage of the first fraction decreased, and changes were observed in the width and position of the second fraction distribution, with the peak shifting towards higher values. For instance, while the particle size distribution for a 2% sample ranged between 1.35 µm (D_10_) and 15.7 µm (D_90_), the distribution for a 10% sample was significantly wider, spanning from 1.79 µm (D_10_) and 93.7 µm (D_90_), accompanied by a noteworthy increase in mean particle size (*p* < 0.05) from 6.4 to 44.3 µm (Table 2). These results support the hypothesis that an increase in extract particle concentration in the oil leads to significant particle aggregation (*p* < 0.05). At higher concentrations, the likelihood of hydrogen bond sites of different particles meeting also increases, contributing to more pronounced particle aggregation and, consequently, the formation of a denser network.

**Figure 4:**
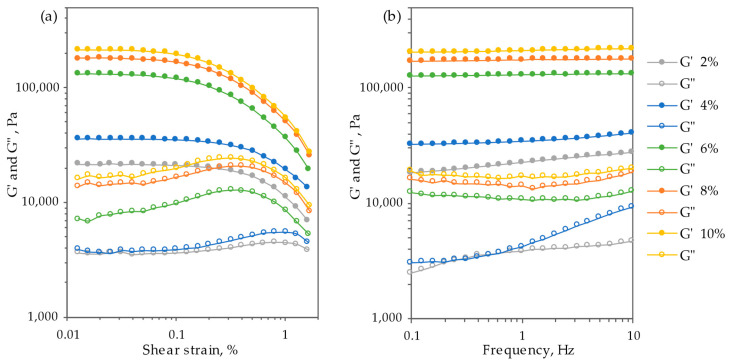
Amplitude sweep (**a**) and frequency sweep (**b**) curves of oleogel samples stabilized with different concentrations of extract particles.

Subsequently, oscillation measurements were conducted to elucidate the structural properties of the obtained oleogels. The results from the amplitude sweeps are presented in Figure 4a. In the linear viscoelastic region (LVR), all samples exhibited a strong gel-like material behavior, the storage modulus (G′) significantly surpassing the loss modulus (G″) by more than a decade. This behavior was further confirmed by the low values of the loss factor tan δ (=G″/G′), which were less than 0.1 in the LVR for samples with an extract particle concentration of 4–10%. The LVR concludes when G′ changes from its plateau value by >5%, a point known as the critical strain. The 2 and 4% oleogels displayed higher critical strain values (0.200 ± 0.011%) than 6, 8, and 10% oleogels (0.081 ± 0.004%). A higher critical strain indicates a wider LVR, suggesting greater resistance to applied force before deformation occurs. Oleogels with particle concentrations of 6, 8, and 10% exhibited lower critical strain and higher G′ values, indicative of a tighter, more brittle network [35,36]. Beyond the LVR, both G′ and G″ rapidly declined as the weak hydrogen bonds in oleogel networks were disrupted. The rheological characteristics of the obtained oleogels are strongly influenced by the extractive particle concentration, with an observed increase in modulus values corresponding to higher particle concentrations. Consequently, an augmentation in particle content leads to an increase in both the material’s consistency and the structuration degree. After defining LVR through an amplitude sweep, the rheological properties of the oleogel structure were further characterized using a frequency sweep (Figure 4b). All oleogel samples exhibited high and nearly constant (very slow slopes) storage modulus values (G′ > G″) throughout the entire frequency range, indicating good tolerance to the time-dependent deformation.

**Figure 5:**
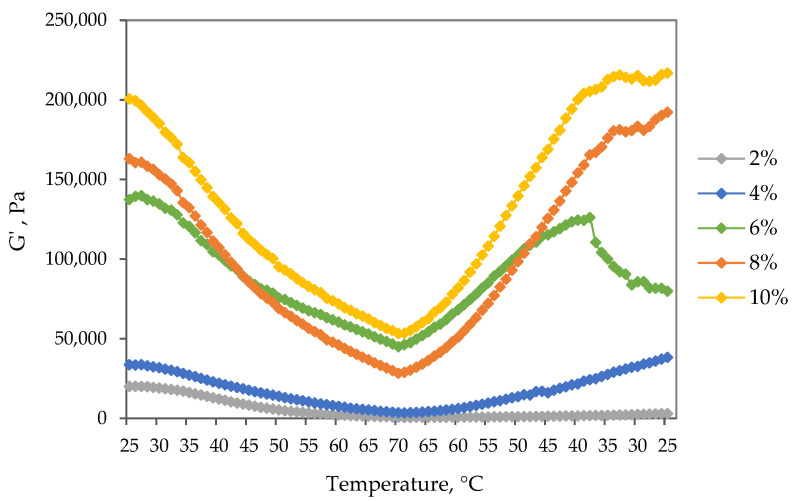
Temperature sweep tests (heating and cooling) obtained for oleogel samples with different extract particle concentrations.

To assess the rheological behavior of oleogels at elevated temperatures, temperature ramp tests under continuous frequency and amplitude were performed. Figure 5 depicts the storage modulus G′ as a function of temperature during both heating (25–70 °C) and cooling (70–25 °C) for oleogel samples with varying particle concentrations. Observations reveal a consistent decrease in the storage modulus values of all oleogel samples with rising temperatures. Initially, at lower temperatures, a slight modulus decrease occurs, but beyond a critical temperature, a notable and relatively sharp decrease is evident, signifying the initiation of system destabilization and network disruption. Despite the continuous decrease in G′ with increasing temperature, a recoverable increase in G′ is observed during temperature reduction, indicating a thermoreversible property. Notably, oleogel samples with particle concentrations of 8% and 10% exhibit full-strength recovery upon cooling. In contrast, the 6% oleogel displays an increase in storage modulus values up to around 40 °C during cooling, followed by a pronounced drop, indicating structural instability. The exception is the sample with the lowest particle concentration of 2%, which lacks structural recovery properties during temperature reduction, maintaining consistently low modulus values.

**Figure 6:**
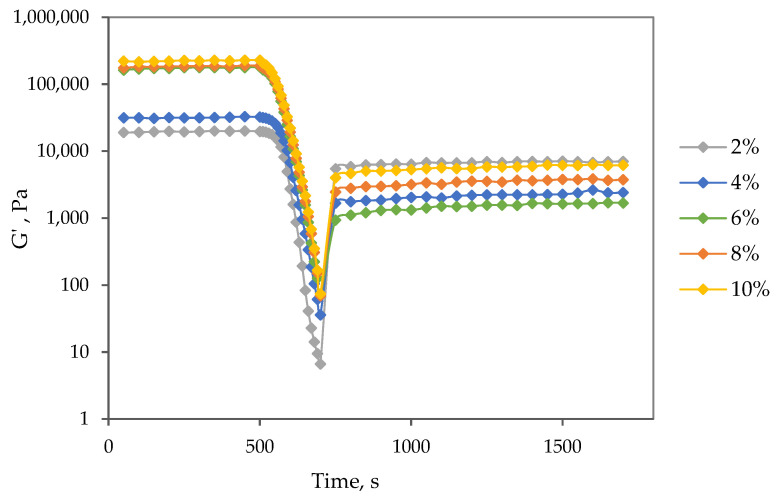
Thixotropic recovery curves of oleogel samples stabilized with different extract particle concentrations.

The impact of time and shear history on the storage modulus of oleogels was investigated through a 3-interval thixotropy test (3-ITT) to comprehend their structure-recovery properties (Figure 6). Initially, at a constant low shear strain, no change in storage modulus values was observed. As the shear strain increased, the structure broke down completely, resulting in storage modulus values dropping to less than 100 Pa. However, during the third interval, as the shear strain was reduced, the samples exhibited partial structure recovery. The sample with the lowest particle content (2%) showed the highest percentage recovery rate at 33.8%. For the 4% gel, the recovery rate was significantly lower at 7.6%, and for gels with higher particle content, the recovery was poor, ranging from 1.0% to 2.7%. These findings suggest that higher gel strength in an oleogel does not necessarily correspond to increased mechanical stability. This observation aligns with a similar report by Martín-Alfonso et al. [37], where a montmorillonite-based oleogel formulated with olive oil exhibited the highest rigidity and the lowest mechanical recovery compared to other vegetable oils.

### 2.2. Effect of Vegetable Oil Type on Oleogel Properties

It has been reported in the literature that each vegetable oil interacts uniquely with the oleogelator, leading to the formation of distinct microstructural gel networks. However, this phenomenon is not fully understood. The interaction between the oil and oleogelator is influenced by factors such as fatty acid profile, molecular weight, and structure of the oil [38]. To assess the gelling properties of birch outer bark extract particles in addition to sunflower oil, we selected three other commonly used vegetable oils (olive, almond, and hemp seed) for oleogel preparation. The major fatty acid compositions of oils used in this study are presented in Table 3. The individual fatty acid content in each vegetable oil varied, resulting in differences in total saturated fatty acids (SFA), monounsaturated fatty acids (MUFA), and polyunsaturated fatty acids (PUFA). Olive oil exhibited the highest level of saturated fatty acids, followed by almond oil, while hemp seed and sunflower oils had comparatively lower levels. Monounsaturated fatty acids were the predominant component in olive, almond, and sunflower oils, while polyunsaturated fatty acids dominated in hemp seed oil. 

Using the selected oils, we successfully obtained stable oleogels with extract particle concentrations of 4, 6, 8, and 10%, which demonstrated stability at room temperature with no signs of oil leakage. However, like sunflower oil, oleogels from other oils with a 2% extract particle concentration exhibited a weak self-standing structure with low storage stability, leading to oil leakage after a few days. Therefore, samples with a particle concentration of 4–10% were chosen for further comparison.

**Figure 7:**
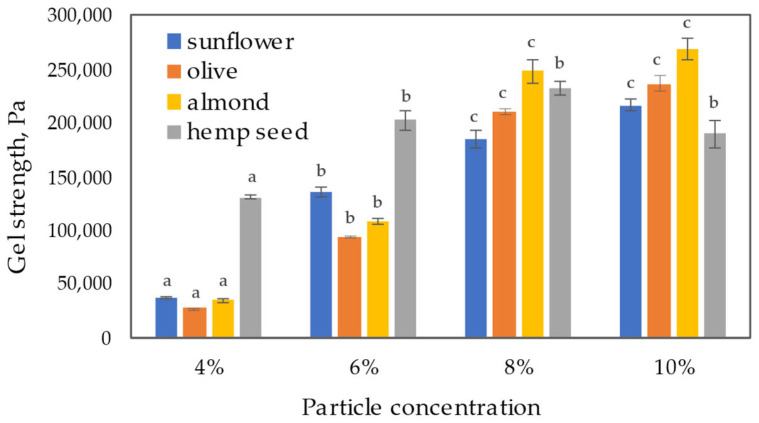
Gel strength as a function of extract particle concentration in gels and oil used. Different letters indicate significant differences among mean values (*p* < 0.05).

The average storage modulus value in the LVR obtained by the amplitude sweep test was used as a gel strength parameter. Comparing oleogels made with different vegetable oils (Figure 7), it is evident that the trends in particle concentration effects were similar. A significant increase in gel strength (*p* < 0.05) was observed with increasing particle concentration up to 8%. With a further increase in particle concentration, the trend of increasing gel strength persisted, but the increase was no longer statistically significant (*p* > 0.05). Hemp seed oil was an exception, where the limit of a significant increase in gel strength (*p* < 0.05) was at a particle concentration of 6%, reaching a maximum of 8%. At extract particle concentrations of 8 and 10%, the almond oil-based oleogels exhibited the highest gel strength values compared to the others, reaching 247 and 268 kPa, respectively. Oleogels from other oils also displayed strong gel properties with gel strength values exceeding 184 kPa. However, at lower extract particle concentrations (4 and 6%), the trend reversed, and hemp seed oil oleogels showed significantly higher gel strength results compared to the other oils (*p* < 0.05), followed by sunflower and then almond and olive oil gels. Martín-Alfonso et al. [37] reported that oleogel structure depends on complex interactions between network structure and oil chemical characteristics. Their study found that vegetable oils with high SFA/MUFA and low UFA/PUFA content form stronger microstructured oleogels. A similar finding was reported by Wang et al. [39], showing that corn oil-based oleogels, due to their higher SFA content, have higher mechanical strength compared to oleogels formulated using linseed and camellia oils. In our case, this trend of the oil effect described in other publications could only be observed at higher extract particle concentrations (8 and 10%) when gels from oils with higher SFA content (olive and almond) showed higher gel strength values. However, at lower particle concentrations, the opposite trend was observed, where hemp and sunflower oil oleogels with lower SFA and higher PUFA content showed higher strength. Generally, the obtained hemp seed oil oleogels showed a lower dependence on gel strength values concerning the concentration of extract particles compared to oleogels of other studied oils. For hemp seed oil oleogels, the difference between the highest and lowest gel strength values in the range of particle concentrations studied was 1.8 times, while for the other oils, this difference was between 6.1 and 8.6 times. The high PUFA content of hemp seed oil may contribute to a more rigid gel structure at a lower particle concentration, and further increases in particle concentration do not show as pronounced an effect as in the case of other oils.

**Figure 8:**
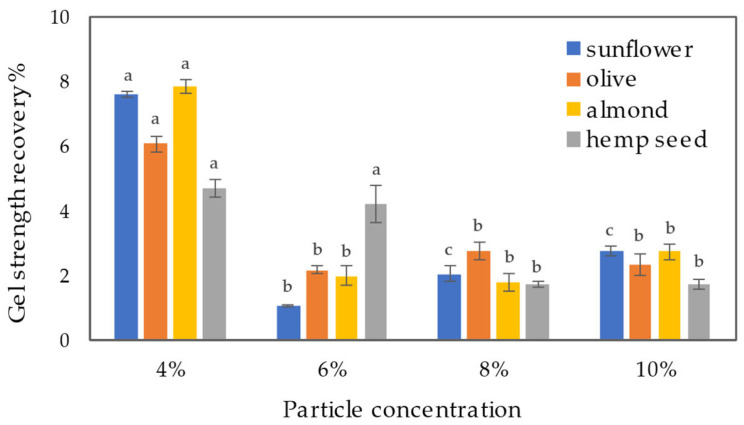
The percentage recovery of gel strength as a function of extract particle concentration and oil used. Different letters indicate significant differences among mean values (*p* < 0.05).

The results of 3-ITT tests, studying the storage modulus of samples over time under alternating constant shear strain, are depicted in Figure 8. Overall, the findings indicate that the obtained samples exhibit significant shear-thinning properties but relatively weak thixotropic properties. Nevertheless, all oleogels samples recovered their gel structure from the liquid sol state when the shear strain was reduced in the third interval of the 3-ITT test. For all oil gel samples, the highest percent recovery was observed at the lowest extract particle concentration of 4%, ranging from 4.7 to 7.8%. Samples with higher particle concentrations (except hemp seed oil at 6%) showed lower percentage recoveries (*p* < 0.05), despite having relatively higher gel strengths at rest. The percentage recovery of the gel strength did not significantly differ (*p* > 0.05) and was below 3%. This behavior is typically associated with brittle gels that display a high gel strength and a narrow LVR [40]. It also aligns with the critical strain values, which for 4% oleogels ranged from 0.159 to 0.201, while for higher particle concentrations, the critical strain values were lower, ranging from 0.079 to 0.101. A gel that exhibits at least 70% recovery after being subjected to a shear stain is considered to have good thixotropic properties [41]. Consequently, the potential use of the obtained oleogels might be limited in applications where a reversible structural regeneration is necessary.

## 3. Conclusions

We successfully produced stable oleogels in various vegetable oils (sunflower, almond, olive, and hemp seed) with particle concentrations ranging from 4% to 10% using a technologically simpler approach. This approach involves direct oleogel production by adding birch outer bark extract particles to the lipophilic phase, combining both solution evaporation and gel formation. The obtained oleogel samples were comprehensively characterized to gain insight into their rheological behavior. A significant effect of the extract particle concentration was observed, where increasing particle concentration led to a proportional increase in gel strength. This phenomenon is attributed to the formation of a more structured network with higher particle concentrations in the oil phase. The resulting oleogels exhibited desirable properties such as good tolerance to time-dependent deformation, shear thinning, and thermoreversibility. However, with regard to the thixotropic recovery, all samples showed poor structure-recovery properties. Furthermore, the type of oil had an impact on the structural properties of the resulting gels. At lower extract particle concentrations (4% and 6%), hemp seed oil oleogels exhibited significantly better gel strength results compared to sunflower, almond, and olive oil oleogels. Additionally, to extend the potential application of extract particles as a gelator, it would be valuable to analyze their capability to structure mineral oils commonly used in cosmetics and pharmaceutical products. Due to the different composition, mineral oils have a lower relative polarity than vegetable oils, which can significantly affect the self-assembly of the particles and the properties of the obtained gels.

Overall, this improved approach for the direct preparation of oleogels from birch outer bark extract solution represents a technologically advantageous step towards the more efficient production of liquid oil-based soft substance systems that can be used in various fields such as food, pharmaceuticals, or cosmetics.

## 4. Materials and Methods

### 4.1. Raw Material

Isolated silver birch (Betula pendula Roth.) outer bark was supplied by a plywood factory (Latvijas Finieris, Riga, Latvia). It was dried at room temperature (moisture content 4–5 wt.%) and milled in an SM 100 cutting mill (Retsch GmbH & Co., Haan, Germany) to pass through a sieve with pores measuring 4 mm in diameter. Next, the milled birch outer bark was fractionated using an AS 200 Basic vibratory sieve shaker (Retsch GmbH & Co., Haan, Germany). A fraction with a size range of 1–3.15 mm was collected and used for further experiments. Sunflower oil (Pernes L, Pernciems, Latvia), olive oil (Medsol srl, Molfetta, BA, Italy), almond oil (Oil Tree, Riga, Latvia), and hemp seed oil (Iecavnieks & Co., Iecavnieki, Latvia) was used as oil phases.

### 4.2. Obtaining Birch Outer Bark Extract

For the extraction process, an average of 3 kg of dried and milled birch outer bark was combined with 18 L of 96 vol.% ethanol (supplied by Kalsnava Distillery, Latvia) in a 30 L reactor. This reactor was equipped with a mechanical stirrer, steam heating jacket, and a reflux condenser. The mixture was stirred and heated to reach the boiling point of the solvent and then was boiled and stirred for 1 h. After that, the solution was filtered hot through a 100 μm filter bag. An additional 5 L of ethanol was used to rinse the extractives from the birch outer bark residues in the reactor. The obtained solution was filtered hot through a 100 μm filter bag and added to the main extract ethanol solution. On average, 20 L of birch outer bark extract ethanol solution was obtained. The dry matter of the extract was 4.51 ± 0.06 wt.%. The basic composition of the dry matter of the birch outer bark extract is shown in Table 4.

### 4.3. Oleogels Preparation

Oleogel samples were obtained according to our developed method [32]. In a 2 L round-bottomed flask, a specific quantity of vegetable oil and a pre-prepared ethanol solution of birch outer bark extract were combined. The flask was then attached to a rotary evaporator (Laborota 4003, Heidolph, Schwabach, Germany) under controlled conditions (bath temperature 50 °C, pressure 100 mbar, rotation speed 80 rpm). The required mass of the oil and the introduced extract solution was calculated depending on the required content of extractives in the oleogel (product mass 100 g). Next, the mixture was distilled until the ethanol residue in the flask was 24 g. The mixture was poured from the flask into a 400 mL beaker and cooled to room temperature. After cooling, the mixture was homogenized with a hand mixer Pro Mix Titanium (Philips, The Netherlands) for 10 s at an average speed of 6000 rpm. To separate the remaining amount of ethanol from the oil, a further evaporation step was carried out at a water bath temperature of 95 °C, a pressure of 20 mbar, and a flask rotation speed of 80 rpm. In the end, the mixture was again poured into a beaker and homogenized with a hand mixer Pro Mix Titanium (Philips, The Netherlands) for 30 s at an average speed of 6000 rpm to maximally disperse the extractive particles in the oil phase. The freshly made oleogel was poured into a Petri dish (14 cm diameter × 2 cm depth) and naturally cooled to room temperature. Obtained oleogels were stored for 7 days at room temperature before further tests to allow the gel network to form after the production process.

### 4.4. Rheological Properties

The rheological properties of oleogels were investigated with the Rheometer MCR 92 (Anton Paar, Graz, Austria) equipped with a 25 mm plate/plate geometry using a gap of 1 mm. Temperature control was provided by a Peltier system and was maintained at 25 °C during the tests. An amplitude sweep test was performed with a logarithmic ramp, a deformation rate of 0.01–100%, at a constant frequency of 1 Hz. The gel strength of the oleogels was expressed as the average value of the storage modulus G′ in the linear viscoelastic region. To determine the mechanical spectra under small amplitude oscillatory shear measurements, a frequency sweep test was performed within the linear viscoelastic region at the frequency range of 0.01–10 Hz. To investigate the structure-recovery properties of oleogels, a 3-interval time test (3–ITT) from Armbruster [43] was used. In this test, the storage modulus G′ of the samples was studied as a function of time under alternating shear strain (0.01, 0.1–100, and 0.01%). To study the temperature behavior, the samples were also subjected to a temperature ramp test (25–70 °C, up and down) at a constant rate of 1 °C/min and frequency of 1 Hz.

### 4.5. Triterpenoid Composition of Birch Outer Bark Extract

To determine the triterpenoid (betulin, lupeol, and betulinic acid) composition of samples, gas chromatography analysis with a flame ionization detector (GC-FID) was performed using a Shimadzu Nexis GC 2030 apparatus (Shimadzu Corporation, Kyoto, Japan). A total of 0.250 mL of pyridine and 0.350 mL of tetrahydrofuran were used to dissolve the samples and analytical standards. The solutions were then heated for 1 h at 70 °C with 0.150 mL of silylation mixture III (N,O-bis(trimethylsilyl)trifluoroacetamide, 1-(trimethylsilyl)imidazole, and trimethylchlorosilane in the volume ratio 3:3:2). A Phenomenex Zebron ZB-35 (30 m × 0.25 mm × 0.25 μm) column was used for the component separation. At 280 °C in a split mode (1:20), the injection volume was 1 μL. The hydrogen flow rate was 32 mL min^−1^, and the detector temperature was 330 °C. Three parallel aliquots of each sample were analyzed by triple injections. Less than 5% varied between the average values.

### 4.6. Fatty Acid Composition of Vegetable Oils

The fatty acid composition of oils was determined by the gas chromatography–mass spectrometry (GC-MS) method. The oils were dissolved in 100 µL of pyridine and 200 µL of silylation reagent and heated at 70 °C for 20 min, then 300 µL of THF was added. A total of 1 μL of the sample was injected into a Thermo Scientific TRACE 1300 gas chromatograph (Thermo Fisher Scientific, Waltham, MA USA) with a Thermo Scientific ISQ quadrupole mass detector. A Thermo Scientific TG-5MS (30 × 0.25 × 0.25) column was used. The injector temperature was 250 °C. In splitless mode, carrier gas (helium) flow was 1.20 mL/min. Oven temperature program: isothermally aged at 150 °C for 5 min, then raised at a rate of 10 °C/min and held for 1 min, finally raised at a rate of 2 °C/min and held at 300 °C for 15 min. The transition line temperature of the mass detector was 250 °C. The ion source temperature was 200 °C. Mass range 45–700 Da.

### 4.7. Fourier Transform Infrared (FTIR) Analysis

The oil and oleogel samples were analyzed with a Nicolet iS50 spectrometer (Thermo Fisher Scientific, Waltham, MA, US) at a resolution of 4 cm^−1^, 32 scans at the 4000–500 cm^−1^ range. The FTIR data were collected using an attenuated total reflectance technique with a diamond crystal prism.

To measure the IR absorption of oil, the IR background spectrum was measured and subtracted. Then, an oil droplet was poured onto the prism. The pouring was carried out so that the oil would cover the entire surface of the diamond crystal prism. Only then were the measurements performed. To measure the IR absorption of oleogel—a soft solid material, first, the IR background spectrum was measured and subtracted. Then, a piece of oleogel was placed on the prism and gently taped so that a contact surface of oleogel and prism would be established. The taping was carried out so that the oleogel would cover the entire surface of the diamond crystal prism. Only then were the measurements performed. All absorption graphs were corrected to have the same baseline.

### 4.8. Particle Size Measurement

The particle size distribution of the oleogels was measured by the laser diffraction instrument Mastersizer 3000 (Malvern Instruments Ltd., Worcestershire, UK) with a Hydro-EV connection. After diluting the sample in a ratio of 1:10 with the oil phase, the sample was added to a glass, where it was stirred in oil to obtain a homogeneous dispersion. The measurement obscuration was set between 3 and 5%, and the stirrer speed was set at 1400 rpm. Laser diffraction results were expressed as D_10_, D_50_, and D_90_ values based on a cumulative volume distribution. The coefficient of variations for the values of five parallel measurements did not exceed 2%.

### 4.9. Statistical Analysis

All tests were performed in triplicate, and the results were expressed as the mean ± standard deviation. The statistical analysis was conducted using Excel data analysis. Anova: Single Factor and Correlation was used to explore the relationships between different variables using *p* < 0.05 as the standard level of significance.

## Figures and Tables

**Table 1:** The basic triterpene composition of birch outer bark extract particles in oleogel.

Total Content of Extractives in Oleogel, wt.%	2	4	6	8	10
Betulin, wt.%	1.01	1.87	2.83	3.77	4.71
Lupeol, wt.%	0.14	0.24	0.36	0.49	0.60
Betulinic acid, wt.%	0.38	0.16	0.24	0.31	0.40

**Table 2:** Particle size data for oleogel samples with different particle concentrations.

Oleogel Sample	D_10_ µm	D_50_ µm	D_90_ µm	D_[4;3]_ µm
2%	1.35 ± 0.01 ^a^	2.1 ± 0.0 ^a^	15.7 ± 0.6 ^a^	6.4 ± 0.2 ^a^
4%	1.44 ± 0.01 ^b^	8.0 ± 0.2 ^b^	32.7 ± 0.6 ^b^	13.1 ± 0.2 ^b^
6%	1.48 ± 0.01 ^c^	11.2 ± 0.2 ^c^	44.8 ± 2.2 ^c^	17.4 ± 0.6 ^c^
8%	1.65 ± 0.01 ^d^	17.0 ± 0.1 ^d^	40.9 ± 1.5 ^d^	21.5 ± 1.2 ^d^
10%	1.79 ± 0.01 ^e^	46.2 ± 0.9 ^e^	93.7 ± 1.7 ^e^	44.3 ± 0.9 ^e^

Different letters in the same column indicate significant differences among mean values (*p* < 0.05).

**Table 3:** The main fatty acid composition of vegetable oils used.

Fatty Acid Species	Fatty Acid Composition (% of Total Fatty Acid)			
	Sunflower Oil	Olive Oil	Almond Oil	Hemp Seed Oil
Palmitic acid (C16:0)	5.79	12.74	7.91	5.90
Stearic acid (C18:0)	3.19	2.78	6.01	2.20
Oleic acid (C18:1)	48.11	74.70	60.34	9.01
Linoleic acid (C18:2)	40.12	6.70	27.19	55.30
Linolenic acid (C18:3)	0.56	0.74	0.79	20.30
SFA	8.98	15.52	13.92	8.10
MUFA	48.11	74.70	60.34	9.01
PUFA	40.68	7.44	27.98	75.60

**Table 4:** The basic composition of dry matter of the birch outer bark extract.

Constituent	Amount, wt.%
Betulin	52.0
Lupeol	7.0
Betulinic acid	2.0
Phenolic compounds	3.4
Unidentified substances *	35.6

* Other terpenoids and their esters, ether oils, hydrocarbons and their epoxides, steroids, tannins, flavonoids, and hydroxycoumarins [42].

## Data Availability

Data are contained within the article.
